# Effect of Cultivation Parameters on Fermentation and Hydrogen Production in the Phylum *Thermotogae*

**DOI:** 10.3390/ijms22010341

**Published:** 2020-12-30

**Authors:** Mariamichela Lanzilli, Nunzia Esercizio, Marco Vastano, Zhaohui Xu, Genoveffa Nuzzo, Carmela Gallo, Emiliano Manzo, Angelo Fontana, Giuliana d’Ippolito

**Affiliations:** 1 Istituto di Chimica Biomolecolare (ICB), CNR, Via Campi Flegrei 34, 80078 Pozzuoli (NA), Italy; mariamichelalanzilli@gmail.com (M.L.); esercizionunzia@gmail.com (N.E.); marco.vastano@gmail.com (M.V.); nuzzo.genoveffa@icb.cnr.it (G.N.); carmen.gallo@icb.cnr.it (C.G.); emanzo@icb.cnr.it (E.M.); afontana@icb.cnr.it (A.F.); 2 Department of Biological Sciences, Bowling Green State University, Bowling Green, OH 43403, USA; zxu@bgsu.edu

**Keywords:** anaerobic bacteria, hydrogen yields, fermentation rate, organic acids, nitrogen, carbon dioxide

## Abstract

The phylum *Thermotogae* is composed of a single class (*Thermotogae*), 4 orders (*Thermotogales, Kosmotogales, Petrotogales, Mesoaciditogales*), 5 families (*Thermatogaceae, Fervidobacteriaceae, Kosmotogaceae, Petrotogaceae, Mesoaciditogaceae*), and 13 genera. They have been isolated from extremely hot environments whose characteristics are reflected in the metabolic and phenotypic properties of the *Thermotogae* species. The metabolic versatility of *Thermotogae* members leads to a pool of high value-added products with application potentials in many industry fields. The low risk of contamination associated with their extreme culture conditions has made most species of the phylum attractive candidates in biotechnological processes. Almost all members of the phylum, especially those in the order *Thermotogales*, can produce bio-hydrogen from a variety of simple and complex sugars with yields close to the theoretical Thauer limit of 4 mol H_2_/mol consumed glucose. Acetate, lactate, and L-alanine are the major organic end products. Thermotagae fermentation processes are influenced by various factors, such as hydrogen partial pressure, agitation, gas sparging, culture/headspace ratio, inoculum, pH, temperature, nitrogen sources, sulfur sources, inorganic compounds, metal ions, etc. Optimization of these parameters will help to fully unleash the biotechnological potentials of *Thermotogae* and promote their applications in industry. This article gives an overview of how these operational parameters could impact *Thermotogae* fermentation in terms of sugar consumption, hydrogen yields, and organic acids production.

## 1. Introduction

The phylum *Thermotogae* is comprised of thermophilic, hyperthermophilic, mesophilic, and thermo-acidophilic anaerobic bacteria that originated from geothermally heated environments (Table 1) [1,2]. Recent phylogenetic analyses based on gene markers/core genome inferences, comparative genomics, and whole-genome relatedness have led to a taxonomic revision of the phylum, with a single class (*Thermotogae*), 4 orders (*Thermotogales*, *Kosmotogales*, *Petrotogales*, *Mesoaciditogales),* 5 families (*Thermatogaceae*, *Fervidobacteriaceae*, *Kosmotogaceae*, *Petrotogaceae*, *Mesoaciditogaceae*), and 13 genera, i.e., *Thermotoga (T.)* [3], *Pseudothermotoga* (*Pseudot.*) [2,4], *Fervidobacterium (F.)* [5], *Thermosipho* (*Ts.*) [6], *Kosmotoga (K.)* [7], *Mesotoga (Ms.)* [8], *Defluviitoga (D.)* [9], *Geotoga (G.)* and *Petrotoga (P.)* [10], *Marinitoga (Mn.)* [11], *Oceanotoga* (*O.*) [12], *Mesoaciditoga (M.)* [13], and *Athalassatoga (A.)* (Table 1) [2,4,14]. *Thermotogae* are able to grow under mesophilic (*Kosmotogales*; *Mesoaciditogales*, *Petrotogales*) and thermophilic conditions (*Thermotogales*), but most species have optimal growth temperatures in the range of 45–80 °C (Table 1). They are Gram-negative bacteria, except for *D. tunisiensis*, which shows a positive result in Gram staining [9]. Apart from *K. shengliensis*, whose cells are in a coccoid form, *Thermotogae* cells are rod-shaped and encapsulated by a unique outer membrane, named “toga” [1,8,15]. Usually, the cells grow singly or in pairs, but it is also possible to observe chains surrounded by a unique toga [1,2]. Cell length is typically less than 20 µm, except for *F. gondwanense* and some members of the *Petrotoga* genus, whose cells can reach to 50 µm long (Table 1) [2,10]. Almost all species grow at neutral pH, and NaCl tolerances are high among *Geotoga*, *Oceanotoga*, and *Petrotoga* species (Table 1). Numerous studies have reported that members of the phylum can grow on both simple (e.g., glucose, galactose, fructose, lactose, maltose, mannose, sucrose) and complex carbohydrates (e.g., starch, glycogen, cellulose, keratin) (Table 1). Genes, transcriptional factors, and regulatory mechanisms driving the carbohydrates utilization have been identified for multiple members of the phylum [16,17,18]. ABC transporters for the uptake of a broad list of sugars have also been characterized [19,20,21,22,23].

All species of the phylum, except for *Mesotoga spp*., have tremendous potentials inbiotechnological production of H_2_, especially the order *Thermotogales*, as their hydrogen yields are close to the theoretical maximum value (Thauer limit) of 4 mol H_2_/mol glucose [1,4,24]. Acetate, lactate, and L-alanine are the major organic products of the sugar fermentation [1]. *Ms. prima* and *Ms. infera* produce mainly/only acetate from sugar utilization without H_2_ formation [8,25,26,27]. Lactate is produced by *T. maritima*, *T. neapolitana*, and *Mn. camini* in variable quantities depending on growth conditions [11,28,29,30,31]. Other significant products include ethanol (has been measured in *Geotoga*, *Petrotoga*, *Kosmotoga*, and *Oceanotoga spp*.); isovalerate, isobutyrate, and/or propionate (have been measured in *Mn. camini* and *K. olearia*); L-glutamate, alpha-aminobutyrate, hydroxyphenyl-acetate, or phenylacetate (have been measured in *F. pennavorans*) [1,32] (Table 1). Among these fermentation end-products, lactic acid has been widely used in various industries such as food, cosmetic, pharmaceutical, and chemical industries, although its primary application is serving as the building block for the production of biodegradable polylactic acid (PLA) [33]. Ethanol is an important industrial commodity; it is used as a food additive and a renewable biofuel; it is also contained in many cosmetics, households, and sanitizer products [34]. Moreover, a plethora of thermostable enzymes, harbored by most of these bacteria, are valuable components for many industrial and biotechnological applications [17,35,36,37,38,39,40,41,42,43,44].

Hydrogen (H_2_) is considered a green and sustainable alternative to traditional fossil fuels and is capable of mitigating greenhouse gas emissions. Using hydrogen in fuel cells or combustion engines produces heat and electricity with water as the only waste. As the current abiotic hydrogen production method is energy-consuming and still causes pollution, emphasis must be given to biological production of the energy from renewable sources [45,46]. Biological synthesis of H_2_ can use a wide range of organic substrates as feedstocks, including agro-industrial wastes and algal biomass, and may operate under various environmental conditions [1,46,47,48,49,50,51,52,53,54]. In addition, high temperatures help to improve the solubilization of substrates, reduce fermentation time, and lower contamination risks [55]. Although hydrogen production by *Thermotoga* species is considered one of the most challenging biological systems, no application using pure *Thermotoga* cultures has been reported at the industrial scale.

Releasing hydrogen is an efficient way to dissipate excessive reductants generated during the fermentative conversion of organic substrates. The process is generally referred to as dark fermentation (DF) and is typically influenced by environmental conditions such as pH, cell growth rate, and hydrogen partial pressure [24,56,57].

**Figure 1:**
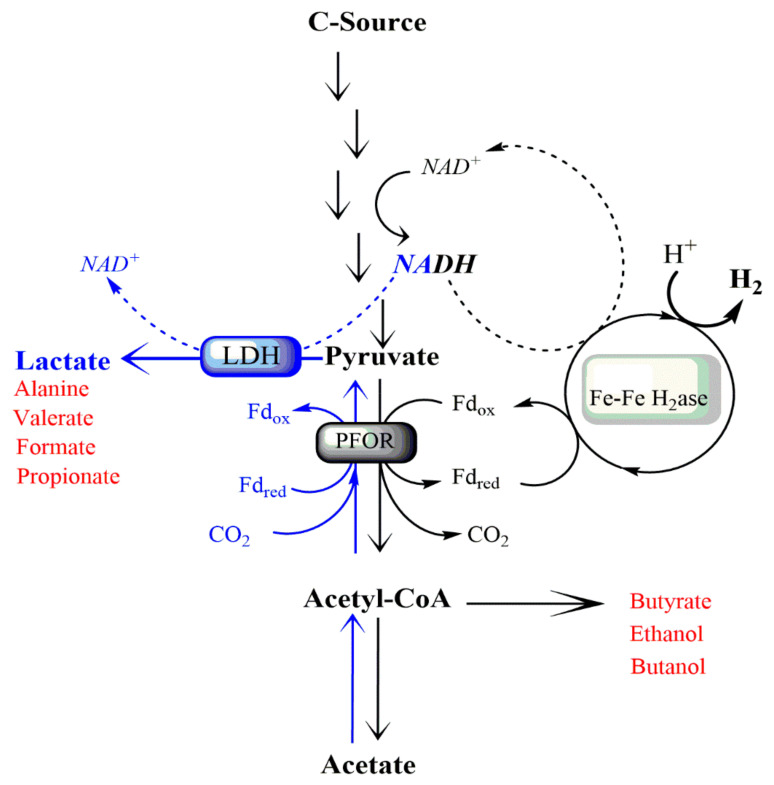
Schematic representation of *Thermotogae* metabolic fermentation. Dark fermentation (black arrows) of glucose leads to the production of H_2_ and acetate. An increase in CO_2_ concentration in the reactor headspace induces the recycling of Ac-CoA and CO_2_ into lactate without impairing the synthesis of biogas (blue arrows). This process is named “Capnophilic lactic fermentation (CLF)” [30,31,56,70]. The main end-products of *Thermotogae* fermentation are H_2_, lactate, and acetate. Other fermentation products are reported in red. Fe-Fe H_2_ase = [Fe-Fe] hydrogenase; PFOR = Pyruvate ferredoxin oxidoreductase; LDH = Lactate dehydrogenase; Fd = Ferredoxin.

According to the classical model of dark fermentation, theoretically up to 4 mol of hydrogen may be produced from each mole of glucose, which is converted to acetate and CO_2_ (Thaeur limit Figure 1) [24]. When hydrogen accumulates, pyruvate is diverted away from acetate production. In this case, excessive NADH from glycolysis is not used in the energetically favorable manner to synthesize acetate and H_2_ but dissipated via synthesizing other metabolic products such as lactic acid, L-alanine, ethanol, butyrate, and valerate (Figure 1) [24]. Synthesis of hydrogen in *Thermotogae* species is performed by the heterotrimeric [FeFe]-hydrogenase, an electron-bifurcating enzyme that couples the endergonic reduction of H^+^ to hydrogen by NADH to the exergonic reduction of H^+^ to hydrogen by reduced ferredoxin (Figure 1) [58]. Because the hydrogenase uses both NADH and reduced ferredoxin as electron donors, hydrogen yield is influenced by factors that affect both reductants.

The value of these bacteria in biotechnological processes is rising sharply since the discovery of the bifurcating hydrogenase and will probably be enhanced with a full elucidation of the molecular and biochemical properties of the processes. Despite decades of efforts in the development of genetic tools to engineer these species, only a few of thermostable selectable markers and genetic modifications with low stability are reported, which makes it still difficult to perform genetic modifications of these organisms [59,60,61]. However, these difficulties could be offset by their well-known susceptibility to mutations under environmental pressures [62,63].

In recent years, many researchers have been focusing on the optimization of fermentation performance towards the production of hydrogen and other target end-products [30,43,64,65,66,67,68,69,70,71].

Anaerobic fermentation in *Thermotogae* depends on many cultivation parameters such as hydrogen partial pressure, agitation, gas sparging, culture/headspace ratio, inoculum, pH, temperature, nitrogen sources, sulfur sources, inorganic compounds, and metal ions. The effect of each factor on H_2_ yield, sugar consumption rate, and formation of biotechnologically interesting end-products are discussed here. Main data are also summarized in extensive tables, citing the most important studies, with the information on their cultivation systems (e.g., reactor type, incubation periods, batch vs. continuous modality).

## 2. Operating Conditions

### 2.1. H_2_ Partial Pressure (P_H2_)

Since *Thermotogae* members are hydrogen producers, tolerance to hydrogen produced by the bacteria on its own gaseous production, known as the “hydrogen partial pressure (*P*_H2_)” effect, is one of the primary parameters being extensively investigated [51,70,105]. The highest hydrogen tolerance has been observed in the genus *Marinitoga. Mn. camini* and *Mn. piezophila* were able to grow with H_2_ concentrations up to 40% and 60%, respectively. *Mn. hydrogenitolerans* and *Mn. okinawensis* can grow under 100% H_2_ atmosphere with only minor inhibition on growth and fermentation [100,101]. Their remarkable resistance to high H_2_ levels is probably related to the typical habitats in which *Marinotoga* species thrive [100]. However, the growth of *Thermotogae* species is often inhibited by H_2_ accumulation, and the metabolism of these organisms undergoes a series of rearrangements to suit *P*_H2_ levels in the bioreactor headspace. The majority of literature data refers to H_2_ percentages in gaseous phase, although some studies have been reporting values of *P*_H2_. Partial pressure around 607 mbar led to decreased levels of biomass production, glucose consumption rate, and H_2_ production in both *T. neapolitana* and *T. maritima* [106,107]. Boileau et al. [107] highlighted a shift of *T. maritima* glucose catabolism from acetic acid towards lactic acid when *P*_H2_ increased from 7 to 607 mbar (Table 2) [106,107]. In contrast, low *P*_H2_ (less than 80 mbar) promoted acetic acid accumulation. Biomass production and glucose consumption rate are unaffected when *P*_H2_ is maintained within the range of 7.1–178.5 mbar (Table 2) [105,106]. In fact, *P*_H2_ lower than 200 mbar is required for optimal growth in reactors, and *P*_H2_ around 2900 mbar completely inhibits growth in *T. maritima* [1,45,49,108,109].

Hydrogen evolution is driven by a bifurcating hydrogenase (H_2_ase) that couples the oxidation of reduced ferredoxin (Fd) and NADH with the reduction of protons to H_2_ (Figure 1) [58]. In dark fermentation, pyruvate is converted to acetate and ATP, which thermodynamically drives the H_2_-acetate pathway. Under high H_2_ partial pressure, hydrogenase activity is inhibited, NADH consumption stops, pyruvate is diverted away from acetic acid production, and lactic acid synthesis becomes the only mechanism for recycling reduced electron carriers (Figure 1) [28,29,30,57,64,106,110]. Synthesis of lactic acid by the lactate dehydrogenase (LDH) catalyzes the conversion of pyruvate to lactate with the concomitant conversion of NADH to NAD+ (Figure 1). The depletion of the pyruvate pool, as occurs with the synthesis of lactic acid, negatively affects hydrogen yield, preventing it from reaching the theoretical maximal value (Figure 1) [24]. This problem can be overcome by enhancing the liquid-to-gas mass transfer and keeping H_2_ concentrations low in experimental conditions (See Section 2.2) or by using mixed cultures with microbial species that are able to oxidize H_2_ [27,111].

### 2.2. Shaking Speed, Culture/Headspace Volume Ratio, Gas Sparging, and Inoculum

Growth and metabolism of thermophilic bacteria are reported to be strongly affected by an increase in the hydrogen level, which makes the metabolic reactions thermodynamically unfavorable [112]. Many effective strategies have been developed to overcome the H_2_ feedback inhibition, such as gas sparging, vigorous stirring, or simply increasing the gas/liquid volume ratio in the reactor. H_2_ saturation is dependent on the partial pressure of hydrogen in the culture medium and its mass transfer from liquid to gas phase. As a matter of fact, the mass transfer of H_2_ from liquid to gas can be improved by applying vigorous agitation in bioreactors [69,106]. Increased H_2_ production rate, glucose consumption rate, and lactic acid synthesis have been observed in *T. neapolitana* cultures with agitation at 200 rpm, compared to static cultures, although the final H_2_ yields were similar [106]. Comparable hydrogen yields were also observed when the agitation speed was 300 and 500 rpm, e.g., 3.0 ± 0.0 mol H_2_/moL glucose at 300 rpm vs. 3.2 ± 0.1 moL H_2_/moL glucose at 500 rpm, with a mild improvement in fermentation rate (Table 2) [69]. In xylose fermentation, the highest hydrogen and organic acid yields have been reported at 400 rpm when tested in the range of 300–600 rpm [113].

To improve hydrogen liquid-gas mass transfer, Dreschke et al. [69] designed a new method that recirculated the H_2_-rich biogas (GaR) into the *T. neapolitana subs. capnolactica* broth with agitation (300, 500 rpm). This combination accelerated the H_2_ evolution rate and glucose consumption rate during glucose fermentation, compared to the treatments including agitation but excluding GaR. Nonetheless, levels of the end-products, except for H_2_ yield, were not significantly altered by the combined parameters (Table 2) [69].

Since *P*_H2_ depends on the culture/headspace volume ratio in the bioreactors, its impacts on the performance of fermentation have also been investigated, mainly in batch reactors. Nguyen et al. [64] have experimented various culture/headspace volume ratio from 8.3% (10 mL/120 mL) up to 50% (60 mL/120 mL) in *T. neapolitana* and *T. maritima* cultures [64]. At 8.3%, the H_2_ production is the highest for both species (890 mL H_2_/L medium in *T. neapolitana* and 883 mL H_2_/L medium in *T. maritima*). H_2_ production gradually diminished, and lactic acid production was promoted with increasing culture volumes [30,64,110]. d’Ippolito et al. [30] found 1:3 culture/headspace volume was the most suitable ratio for high hydrogen yields [30]. When these conditions were optimized, *T. neapolitana* resulted in H_2_ yields between 3.46–3.85 mol H_2_/mol glucose [30,114].

Gas sparging, mainly with N_2_, is the most common method to reduce hydrogen partial pressure by removing H_2_ and CO_2_ produced from sugar fermentation in closed bioreactors [56,108,115,116]. Under nitrogen sparging conditions, the overall yield of H_2_ in *T. neapolitana* fermentation was about two-fold of the non-sparged cultures, e.g., 1.82 vs. 3.24 moL H_2_/moL glucose or 1.14 vs. 2.20 moL H_2_/moL xylose (Table 2). The levels of acetic acid and butyrate also increased [110]. Moreover, the fermentation performance was remarkably improved when N_2_- sparging was coupled with pH control in *T. neapolitana* using pure glycerol as the sole carbon source (Table 2) [116]. Keeping pH close to neutral improved the glucose utilization and H_2_-acetate production rates. In contrast, lactic acid production was lowered under these conditions (0.255 mmol/L with pH control and sparging vs. 0.36 mmol/L with pH control but no sparging) (Table 2) [116]. The use of a CO_2_-enriched atmosphere significantly increased both glucose consumption rate and hydrogen production rate, even though the molar yield was comparable to that of N_2−_sparging (Table 2) [31]. Surprisingly, supplementation of CO_2_ to *T. neapolitana* cultures induced an unexpected metabolic shift from acetic to lactic fermentation without any significant change in hydrogen production (3.6 moL/moL glucose) (Table 2) [31]. Experiments with labeled precursors revealed that part of the exogenous CO_2_ was biologically coupled with acetyl-CoA to give lactic acid when the cultures were sparged with CO_2_ gas or enriched in sodium bicarbonate (Figure 1) [117]. This process, named Capnophilic Lactic Fermentation (CLF), has the surprising feature to produce more lactic acid than expected from the classical dark fermentation model where H_2_ production is impaired by the onset of by-passing pathways (Figure 1) [31,56,117,118,119]. In dark fermentation, hydrogen and lactic acid levels competed for a common pool of reducing power. Whereas, in CLF, the H_2_ level remained high, probably due to additional sources of reductants to sustain NADH-dependent pathways (Figure 1) [118,119,120]. Recently, an additional increase in lactic acid production occurred in a *T. neapolitana* mutant that was isolated from a culture adapted to continuous exposure to CO_2_ [62]. Sparging with CO_2_ was also performed on the culture of other *Thermotogales* species, whose metabolic response was qualitatively and quantitatively diverse (Table 2) [70]. CO_2_-enriched conditions promoted glucose consumption rate and lowered biogas production in almost all tested species [70]. *T. caldifontis*, *Pseudot. elfii*, *Pseudot. thermarum*, *Pseudot. lettingae*, and *Pseudot. subterranea* did not show substantial variations in the levels of the fermentation products compared to cultures in an N_2_-enriched atmosphere [70]. *T. neapolitana*, *T. maritima*, *T. profunda*, and *Pseudot. hypogea* species responded to CO_2_ by reducing the fermentation rate. *T. neapolitana subsp. capnolactica* was the only species to increase lactic acid and H_2_ yield moving from N_2_-sparging to CO_2_-sparging [70]. Generally speaking, the supplementation of external gas (N_2_ or CO_2_) successfully improves the fermentation performance in most species and lowers the inhibitory effect of H_2_ accumulation, but it inevitably causes an undesired dilution of hydrogen in evolved gases. In this context, the recirculation of the H_2_-rich biogas method prevents hydrogen saturation in the bioreactor without negatively affecting the content of the produced biogas [69].

The initial biomass concentration (size of inoculum) also has an unexpected impact on the fermentation of thermophilic bacteria. Using various initial biomass concentrations of *T. neapolitana subs. capnolactica* (in the range of 0.46–1.74 g CDW/L) under CO_2_ atmosphere, hydrogen yield and the distribution of end-products were unaffected (Table 2) [68]. However, increasing inoculum size from 0.46 to 1.74 g/L reduced the fermentation time from 7 h to 3 h [68]. Moreover, the hydrogen production rate, glucose consumption rate, and biomass growth rate were increased [49,50,68]. It is worth pointing out that Ngo et al. [116] reported a reverse correlation between hydrogen production rate and inoculum size, stating that high initial biomass corresponded to a mild reduction of hydrogen production rate [116].

### 2.3. pH

As the fermentation of sugars leads to the production and accumulation of organic acids, the pH is decreasing during the process, which may inhibit bacterial growth before the substrates are completely consumed [30,106,113]. Two factors impose a strong inhibition on bacterial growth and H_2_ production: rapid decrease in pH due to the accumulation of byproducts and feedback inhibition caused by H_2_ accumulated in the headspace [65,105,106,107,108,113,121].

Thus, pH is a critical factor to control sugar consumption and direct end-products formation [65,67,117,119,122]. Gradual pH drop causes enzyme activity loss [123]. To overcome pH-induced limitations on *Thermotogae* fermentation, several studies were performed with pH adjustments [51,67,121]. In pH-controlled cultures (~6.5–7.0), H_2_ and acetic acid production predominated over lactic acid and peaked around 20 h [113]. In contrast, lactic acid production only started when pH declined to around 5.0 [113].

The addition of NaOH at regular intervals and the use of buffering reagents have been regarded as the best-performing methods with serum bottles [56,66,67,113]. The optimum pH for growth and hydrogen production is 6.5–7.0 in *T. maritima* and 6.5–7.5 in *T. neapolitana* depending on substrates and growth conditions [64,113,122]. Moreover, pH 7.0 provides the most promising results in terms of H_2_ and organic acids production in *T. neapolitana* [113,122]. A pH shift from 5.5 to 7.0 improved H_2_ yield from 125 to 198 mL H_2_/L medium in *T. neapolitana* [61]. With *T. neapolitana* cells immobilized on ceramic surfaces using glucose as the carbon source, the highest hydrogen production was observed in the pH range of 7.7–8.5 [51]. Further increase in the range of pH to 8.0–9.0 led to a dramatic decrease in the biogas evolution [64].

Different organic and inorganic buffers have been examined for their effect on anaerobic fermentation under various growth conditions and buffer concentrations [51]. According to Cappelletti et al. [51], 0.1 M HEPES resulted in the best performance, compared to MOPS, PIPES, HPO_4_^−^/H_2_PO_4_^−^, or Tris-HCl buffer in *T. neapolitana* batch cultures growing on glucose under N_2_ atmosphere [51]. The good buffering properties of HEPES, whose pK (7.55) is near the optimal pH of *T. neapolitana,* was also demonstrated for *T. neapolitana* cultures growing on different complex carbon sources (cheese whey, molasses, or waste glycerol) [51,122]. In another study, 0.05 M HEPES was found to be sufficient under N_2_ sparging atmosphere (Table 2) [113]. Under CLF conditions, 0.01 M MOPS, TRIS, or HEPES buffers provided satisfactory results for both H_2_ and lactic acid synthesis in *T. neapolitana subs. capnolactica* (Table 2) [67]. More specifically, H_2_ synthesis was found to be the highest in MOPS, while TRIS promoted acetic acid formation (Table 2) [67]. The highest value of lactic acid synthesis was 14.9 ± 0.3 mM in phosphate buffer compared to 11.3 ± 0.6 mM in the standard condition (Table 2) [67].

The buffering capacity of HCO_3_^−^ is sufficient to maintain near to optimal pH for growth (~6.5), facilitating the complete substrate degradation and desired by-product formation (Table 2) [31,56,67].

In other studies, itaconic acid was successfully used as a physiological buffer to enhance hydrogen production in *T. neapolitana* growing on glucose or glycerol [121,122]. During the cultivation with 1.5 g/L itaconic acid, the pH slowly dropped from 7.5 to 6.8 over 99 h, while the same pH change was reached within 48 h in cultures not buffered [122]. Although itaconic acid is only poorly catabolized, it affected the overall metabolism of *T. neapolitana* because H_2_ and acetic acid production were almost 1.4-fold higher than the control, while lactic acid production was reduced by nearly 100% compared to the control (Table 2) [122]. In addition, Ngo and Sim [122] found that the performance of *T. neapolitana* fermentation growing on waste glycerol was improved by almost 40% by adding itaconic acid into the culture medium [122].

### 2.4. Temperature

Due to their origin from hot habitats, bacterial species of the phylum *Thermotogae* can live and grow at temperatures in the range of 40–90 °C (Table 1). Some species such as *K. olearia*, *O. teriensis*, *Ms. prima*, and *P. mexicana* can thrive at mesophilic temperatures (Table 1) [7,8,96,100], and other species such as *F. changbaicum*, *F. thailandese*, *T. maritima*, *Pseudot. hypogea*, and *T. neapolitana* share the ability of growing at temperatures close to 90 °C (Table 1) [3,74,77,83,94]. For a long time, researchers have selected an operating temperature of 70 °C [104,117] or 80 °C [105] to cultivate *T. neapolitana* and *T. maritima* without careful investigation of the impacts on fermentation. Nguyen et al. [64] explored changes of H_2_ production with temperatures ranging from 55 to 90 °C for *T. neapolitana* and *T. maritima*. Both cultures showed approximately 100 mL H_2_/L medium at 55 °C and a maximum of 200 mL H_2_/L medium at 75–80 °C, with a decrease to 150 H_2_/L medium at 90 °C [64]. In *T. neapolitana*, high temperatures (77–85 °C) enhanced glucose uptake (2.2 mmol/L at 60 °C and 11.0 mmol/L at 77–85 °C) and boosted hydrogen yields (2.04 mol H_2_/moL consumed glucose at 60 °C and 3.85 mol H_2_/mol at 77 °C) [65]. This positive effect was also found for acetic acid (2.0 mmol/L at 60 °C and 18.0 mmol/L at 85 °C) and lactic acid production (no production at 60 °C and 1.25 mmol/L at 85 °C) (Table 2) [65]. Studies conducted on *T. maritima* hydrogenase demonstrated that this enzyme is unstable at the ambient temperature and its activity increased considerably with rising temperature (an activity of 25 units/mg at 20 °C and 110 units/mg at 90 °C [123].

### 2.5. Oxygen (O_2_)

*Thermotogae* members occur in various hot ecosystems, including hot springs, deep-sea, and shallow hydrothermal vents, and may also be exposed to O_2_ in these ecological niches [1254]. Indeed, despite their anaerobic nature, O_2_ tolerance is variable in the phylum; for example, *Thermotoga*, *Fervidobacterium*, and *Geotoga* genera can grow only under strictly anaerobic conditions, while *K. olearia* can survive in up to 15% O_2_ [10]. With elemental sulfur, *Ts. atlanticus* can grow with up to 8% O_2_ in the headspace [92]. Geochemical and microbial analyses demonstrated the wide distribution of *Thermotogae* species in ecosystems that are not only anaerobic but also partially oxygenated [124]. For this reason, the question of O_2_ tolerance and microaerophilic metabolism of *Thermotogae* has been addressed by several studies [65,105,106,125,126,127,128,129]. Some researchers have demonstrated that low concentrations of O_2_ are tolerated by *T. neapolitana* and *T. maritima* [127,128]. An O_2_ insensitive hydrogenase has been described in *T. neapolitana*, explaining why microaerobic H_2_ production and O_2_ tolerance could take place in this bacterium [130]. Additionally, *Pseudot. hypogea* and *T. maritima* contain an NADH oxidase that may serve as an O_2_ detoxification system [131,132]. Lakhal et al. [129] demonstrated O_2_ consumption over 12 h during the stationary phase of *T. maritima* in a batch reactor without reducing agent [129]. O_2_ presence reduced glucose fermentation rate and significantly shifted metabolism towards lactic acid production in *T. maritima* (Table 2). This change can probably be explained by O_2_ sensitivity of the hydrogenase [129]. Furthermore, *T. maritima* overproduced enzymes involved in reactive oxygen species (ROS) detoxification, iron-sulfur cluster synthesis/repair, cysteine biosynthesis, and a flavoprotein homologous to the rubredoxin of *Desulfovibrio* species that exhibited an oxygen reductase activity [127].

Van Ooteghem et al. [121] reported that O_2_ concentration decreased during the growth of *F. pennavorans*, *P. miotherma*, *Ts. africanus*, *Pseudot. elfii*, and *T. neapolitana*. In these experiments, the H_2_ yield greatly exceeded the theoretical limit of 4 mol H_2_/mol glucose in *F. pennavorans*, *Pseudot. elfii*, and *T. neapolitana* fermentation [121]. These surprisingly high H_2_ yield have led to the hypothesis of an unidentified aerobic pathway using O_2_ as a terminal electron acceptor in these bacteria which may not be obligate anaerobes [121]. However, aerobic metabolism is not supported by the genomic sequence of *T. maritima*, although the enzymes involved in the pentose phosphate pathway and an NADPH-reducing hydrogenase have been identified in the genome [16]. To explain the increased yield of H_2_ by *T. neapolitana* in microaerobic conditions and the existence of a catabolic process requiring O_2_, van Ooteghem et al. [121] used malonic acid as an inhibitor of succinate dehydrogenase and thus the O_2_-dependent metabolism. Even if the coding sequence for succinate dehydrogenase has not been identified in the *T. maritima* genome, hydrogen generation was completely inhibited for >40 h in the presence of malonate, postulating that malonate in the medium was no longer available to block catabolism [121]. Then, Eriksen et al. [106] demonstrated that malonic acid was not metabolized by *T. neapolitana* cultures but the exposure to malonic acid clearly affected the metabolism as reduced production of lactic acid and increased H_2_ yield were observed [106]. Against these findings, other researchers reported a reduction of H_2_ rate and production in *T. neapolitana* cultures after the injection of 6% O_2_ [65,106]. The reduction of O_2_ consumes reducing equivalents that are then unvailable to produce H_2_. The total duration of *T. maritima* fermentation in the batch reactor was delayed about 67 h under O_2_-induced stress [129]. In addition, the consumption rate of glucose was drastically reduced and the metabolism of *T. maritima* shifted towards lactic acid production due to inhibition of the O_2_-sensitive hydrogenase [129].

From a technical point of view, several strategies were adopted to remove dissolved O_2_ in the bioreactor: [I] sparging the culture with N_2_, CO_2_ or a mixture of both gases; [II] heating the medium; [III] adding a reducing agent such as sodium sulfide or cysteine-HCl in the medium; [IV] maintaining a positive pressure in the bioreactor headspace [31,56,62,67,70,105,106,113,121].

## 3. Nitrogen Containing-Compounds

Nitrogen sources (N-sources) are essential for bacterial life for the synthesis of cellular components like nucleic acids, proteins, and enzymes [133,134]. Yeast extract (YE), tryptone, and ammonium chloride (NH_4_Cl) have been identified as highly efficient and versatile organic N-sources in laboratory practices. It is widely demonstrated that most of the *Thermotogae* members can use yeast extract and tryptone to grow and metabolize carbohydrates [1,10,77,108,135,136].

Numerous efforts were made to replace YE by combining casamino acids and amino acids, but *Pseudot. elfii* failed to grow on these alternative substrates. The biogas yields of cultures grown with other N-sources were about 4–14% of those with YE (Table 3) [108].

Experiments with different concentrations of YE and tryptone were performed to identify their optimal and minimal concentrations in growth media [64,108,122,137,138]. YE and tryptone are sufficient to ensure growth and hydrogen production without additional carbon sources in *Pseudot. elfii* (Table 3) [108]. van Niel et al. [108] used media with various concentrations of YE and tryptone to ferment glucose by *Pseudot. elfii* [108]. They discovered that increasing the contents of both YE and tryptone from 2 g/L to 5 g/L improved H_2_ production (14.8 vs. 28.8 mmol/L) but higher contents did not further improve hydrogen and acetic acid production; high levels of both YE and tryptone only increased acetic acid production in medium lacking other C-sources [108].

When there was a low level of YE (2 g/L) but no tryptone, productions of H_2_ and acetic acid remained low, suggesting that tryptone served as an energy source like YE (Table 3) [108]. Although the amino acid compositions of the two N-sources are fairly similar, tryptone contains abundant peptides, a preferred form of amino acids by many bacteria [138]. In another study [122], *T. neapolitana* biomass increased along with the increase of YE concentrations in the range of 1.0–4.0 g/L but not with higher YE concentrations (5.0–6.0 g/L) [122]. The H_2_ production plateaued at 420 mL/L in *T. neapolitana* growing on glycerol with 1.0–4.0 g/L YE [122]. Experiments in *T. maritima* and *T. neapolitana* revealed that with over 2 g/L YE, there was a clear increase of acetic acid production, and hydrogen counted up to 30-33% of the total gas in the headspace, even though a mild reduction in glucose consumption occurred (Table 3) [64,138].

Nevertheless, low concentrations (2–4 g/L) of YE are still able to support productivity and bacterial growth [64,108,122,138]. d’Ippolito et al. [30] reported that 2 g/L of both tryptone and YE contributed to 10–15% of the total fermentation products in *T. neapolitana* [30]. Balk et al. [75] demonstrated that *Pseudot. lettingae* was able to degrade methanol in around 30 days in the presence of 0.5 g/L YE, whereas the substrate degradation did not occur when YE was omitted [75]. In contrast, the fermentation of *T. neapolitana* with glucose occurred in a medium without YE, even though the total glucose consumption without YE was attained in 30 h rather than 12 h. H_2_ and acetate amounts were half in the medium without YE, (Table 3) [135].

The impact of an inorganic N-source on *Thermotogae* fermentation, such as NH_4_Cl, has not been extensively studied, but the presence of NH_4_Cl has often been associated with either exopolysaccharide (EPS) formation in *T. maritima* or alanine production in *T. neapolitana* [62,129,136,139]. It is not clear how NH_4_Cl stimulates EPS production, but it might involve processing the surplus of reducing equivalents. For example, some organisms produce EPS as a mechanism to transport reducing equivalents out of the cell [140].

Han and Xu [61] demonstrated that a surplus of NH_4_Cl could partially substitute YE and tryptone in an optimized medium for auxotrophic *Thermotoga* sp. *RQ7* strain [61].

## 4. Sodium Chloride and Phosphate

All members of the phylum *Thermotogae* showed great adaptability to a wide range of salinity levels (Table 1), although the optimal concentrations of NaCl vary among the members. *Geotoga*, *Oceanotoga*, and *Petrotoga* species can survive in environments comprised of 10% NaCl, while *P. mexicana* can live in up to 20% NaCl (Table 1) [10,12,95]. In contrast, species of the genus *Fervidobacterium* can tolerate salt concentrations up to 1% [5,79,80,81,83]. Among the species of the genus *Mesotoga*, *Ms. infera* exhibited the lowest tolerance of NaCl (Table 1).

NaCl at 20 g/L was reported to be optimal for *T. neapolitana* growing on either glucose or glycerol when hydrogen production is concerned [64,105,106,108,110,116]. Recently, the effect of different NaCl concentrations (0–35 g/L) on the CLF process was explored in *T. neapolitana subs. capnolactica* using glucose as the carbon source [67]. H_2_ synthesis and biomass growth were reduced by 15% and 25%, respectively, when NaCl was increased to 35 g/L (Table 3). Similarly, acetic acid production decreased from 26.1 ± 4.7 mM with 10 g/L NaCl to 23.2 ± 0.8 mM with 35 g/L NaCl. In contrast, high NaCl levels had a positive impact on lactic acid production, which increased 7.5-fold (2.8 ± 0.3 mM at 0 g/L NaCl vs. 21.6 ± 6.2 mM at 35 g/L NaCl), without affecting the overall H_2_ yields (Table 3) [67]. Pradhan and coworkers [67] suggested a possible involvement of NaCl in a sodium ion gradient that potentially fuels ATP synthesis and transport processes [67]. This creates a bioenergetic balance and supplies necessary reducing equivalents to convert acetic acid into lactic acid under CLF conditions (Figure 1) [67,118,119]. Similarly, another study [141] on H_2_-producing *Vibrionaceae* showed that increasing NaCl levels from 9 to 75 g/L enhanced lactic acid synthesis [141].

Regarding phosphate species, they have a strong buffering ability to mitigate pH fluctuation caused by the accumulation of volatile fatty acids [142]. Phosphate deficiency induced an increase in lactic acid production and a small decrease in H_2_ formation, suggesting a slight shift of the *T. maritima* metabolism towards lactic acid production. Besides its role as a macro-element, phosphate can also interact with calcium, favoring H_2_ production [141,143]. Saidi and co-workers [52] showed that *T. maritima* struggled to produce H_2_ at the same rate when there was an oversupply of calcium but an undersupply of phosphate in the medium [52]. For unknown reasons, phosphate exceeding 50 mM has been suggested to inhibit *Pseudot. elfii* growth [108].

## 5. Sulfur-Containing Compounds

All members of the phylum *Thermotogae* reduced sulfur-containing compounds such as elemental sulfur (S^0^), thiosulfate (Thio), and polysulfide to hydrogen sulfide (H_2_S), which is produced at the expense of H_2_ (Table 1) [1,4,29,76,144,145]. Sufficient supply of sulfur-containing compounds seems to be critically important; due to a large requirement for Fe-S clusters by the hydrogenase (containing 20 atoms of Fe and 18 atoms of S), PFOR, and other enzymes (Figure 1) [123,146]. In the literature, the effect of sulfur sources has been widely explored. The reduction of S-sources is considered an electron-sink reaction to deplete the surplus of electron power [3,98,107,147]. It is well known that the growth of most anaerobic bacteria of the phylum *Thermotogae* is stimulated by S-sources, but not dependent on them [1,29,52,53,75,107,125,126,144]. Generally speaking, the substrate consumption rate is benefited from a sulfur supply in the medium, except for the methanol fermentation in *Pseudot. lettingae,* which is reduced by S-containing compounds (19.7 mmol/L w/o S-source, 18.7 mmol/L with Thio and 10.6 mmol/L with S^0^) (Table 4). Members of the *Mesotoga* genus are able to oxidize sugars, although with low efficiency, only when S^0^ is used as the terminal electron acceptor [26,27,66,148,149]. This process gives acetic acid, CO_2_, and sulfide (2 mol of acetate and 4 mol of sulfide per mol of glucose), with no or trace amounts of H_2_ (Table 4) [27]. After 250 days of *Ms. prima* cultivation, 9.21 ± 0.13 mmol/L of acetate was measured in the presence of S^0^ rather than 1.67 ± 0.21 mM obtained in its absence (Table 4) [27]. Fadhlaoui and collaborators [27] argued that the metabolic differences between *Thermotoga* spp. and *Ms. prima* strains are related to the absence of a bifurcating [FeFe]-hydrogenase and the accumulation of NADH in *Ms. prima*, leading to growth inhibition in the absence of an external electron acceptor [27]. However, *Ms. prima* and *Ms. infera* strains grew more efficiently in a syntrophic association with a hydrogenotrophic microbial partner that serves as a biological electron acceptor compared to growing *Mesotoga* in a pure culture with sulfur as electron acceptor [26,27]. Boileau et al. [107] investigated the different responses of fermentation performance to different S-sources (Table 4) [107]. Among these compounds (Table 4), thiosulfate, cysteine, and Na_2_S were the most efficient ones to optimize *T. maritima* glucose fermentation (Table 4) [107]. Biogas production and glucose utilization increased in the order of no S-source < DMSO < S^0^ < Thio < Methionine (Met) < Na_2_S < Cysteine (Cys) (Table 4) [107]. Moreover, Na_2_S and Cys increased acetic acid production 3-fold and H_2_ production 2-fold (Table 4). Thiosulfate seemed to promote lactic acid formation (0.8 ± 0.1 mM w/o S-source and 6.3 ± 0.6 mM with Thio) without affecting other products [107]. Surprisingly, lactic acid was dependent on thiosulfate concentration (0.3 mol/mol glucose w/o Thio and 0.6 mol/mol glucose with 0.24 mmol Thio), even though the proportion between lactic and acetic acid yields remained constant (Table 4). DMSO had no significant impact on *T. maritima* fermentation parameters (Table 4) [107].

In the presence of thiosulfate, the growth and glutamate production of *Fervidobacterium* is stimulated; however, S^0^ does not seem to help overcoming the H_2_-feedback inhibition (Table 4) [32,80,88,144]. *P. olearia*, *P. sibirica*, and *Ts. Africanus* produced small amounts of ethanol (0.17 mM for both *Petrotoga* species and 0.79 mM for *Ts. africanus*) only in the absence of S-sources (Table 4) [93,145]. *Pseudot. lettingae* produced L-alanine, at the expense of acetic acid, only when thiosulfate or S^0^ was present in the medium using methanol as the substrate (Table 4) [75]. Meanwhile, the presence of thiosulfate or S^0^ resulted in increased production of acetic acid and decreased production of alanine in *Pseudot. hypogea*, *Ts. melaniensis*, *Ts. geolei*, *P. olearia*, and *P. sibirica* cultures, using glucose or xylose as the carbon source (Table 4) [77,87,90,93]. When S^0^ is available, no hydrogen could be detected in *Mn. hydrogenitolerans* growing on glucose [101].

*Thermotogae* members have been widely employed to degrade different organic wastes, and their degradation significantly benefited from the presence of a reducing agent [51,52,53,54,113,116,138]. It is noteworthy to mention that high concentrations of thiosulfinate, a volatile organo-sulfur compound found in organic wastes, has an inhibitory effect on *T. maritima* growth [54]. Similarly, Tao et al. [150] demonstrated that thiosulfinate inhibited the H_2_ production by mesophilic seed sludge when co-fermenting food wastes [150].

## 6. Metal Ions

Typically, hydrothermal ecosystems are enriched with essential micronutrients and trace metals such as soluble and insoluble iron, manganese, cobalt, and molybdenum. Some terrestrial hydrothermal waters are also characterized by chromium and uranium contents of several micrograms per liter [151]. The physiological roles that most of these metals play in microbial metabolism are still largely unknown. It is believed that their functions include energy generation and biosynthesis [151]. In addition, Mn, Fe, Zn, and Co metals are vitally important micro-elements for growth, essential for cellular transport processes, and serve as cofactors for many enzymes [152]. Understanding the physicochemical properties of extreme habitats can help to determine the metal toxicity limits on microbial growth in laboratory settings. Indeed, metal susceptibility tests have been carried out on *T. neapolitana*, *T. maritima*, and *Ts. africanus*, and have identified the following toxicity order: cadmium (1.0–10.0 µM) > zinc (0.01–0.1 mM) > nickel (1.0–5.0 mM) > cobalt (1.0–10.0 mM) [153].

Attention has also been paid to Fe (III) reduction by thermophilic bacteria, since Fe (III) may work as an external electron acceptor in microbial metabolism [154]. Members of the phylum *Thermotogae* are capable of coupling the reduction of iron with the oxidation of a wide range of organic and inorganic compounds. *T. maritima* reduced Fe (III) into Fe (II) exclusively with molecular hydrogen as an electron donor [154]. Fe (III) reduction has also been reported to stimulate growth and mitigate H_2_ inhibition in *Pseudot. lettingae*, *Pseudot. subterranea*, *Pseudot. elfii*, *Ts. affectus*, *Ts. globiformans*, and *Ts. activus* [75,76,88,89,91]. The recently characterized member of the order *Mesoaciditogales*, *A. saccharophila*, changed fermentation end-products when growing with Fe (III), favoring the production of small amounts of acetate, isobutyrate, and isovalerate [14].

Ions and metals are generally supplied in *Thermotogae* growth media through Balch’s oligo-elements solution [155]. The removal of oligo-elements from *T. maritima* cultures resulted in a minor increase in lactic acid production (1.2 vs. 4.3 mmol/L) and a decrease in H_2_ productivity (12.4 vs. 8.8 mmol/h/L) [52]. Limitation in iron lowered H_2_ production by deviating the fermentation pathway towards the production of more reduced end-products such as lactic acid in mixed cultures [156,157]. Another study [139] highlighted how the supplementation of Fe ions to mixed cultures had pronounced effect on hydrogen activity [139]. Similarly, Fe^2+^ (as well as Co, Ni and Mn) stimulated *Pseudot. hypogea* alcohol dehydrogenase activity (ADH), an iron-containing enzyme involved in alcohol fermentation, by 10–15%, while Zn^2+^ completely inhibited the enzyme activity [158]. On the same base, the inclusion of tungsten in the growth medium of *T. maritima* increased the specific activity of both hydrogenase (by up to 10-fold) and PFOR in cell-free extracts, although the function of tungsten in the metabolism of *T. maritima* is not clear [123,126].

As for magnesium, potassium, and calcium ions, they not only play critical roles in bacterial growth, but also act as enzyme cofactors and ensure the survival of microorganisms in their hot ecosystems, by protecting double-stranded DNA from degradation [159]. The best cell yields were obtained with a low concentration of Mg^2+^ and a high concentration of Ca^2+^ [126]. It would be worthwhile to dig further into the metal ions repercussions on *Thermotogae* metabolism in future research.

## 7. Conclusions

Steam reforming of methane (CH_4_) is currently used to produce hydrogen in the industry, as it is the most economic technology available so far. Producing hydrogen by biological means at an industrial scale remains as a challenge. Within the race to find the best way to generate hydrogen via microbes (e.g., choice of strains, substrates, fermentation conditions), *Thermotogae* seem to have many unique advantages. Optimization of their cultivation conditions is fundamental to improve the overall productivity of the fermentation system and its profitability, which determine the feasibility of replacing the current methods of hydrogen production.

The phylum *Thermotogae* comprises a wide collection of species with astonishing and unique features associated to their original habitats. Extensive research has shown tremendous potentials of using these bacteria in biological production of hydrogen, degradation of wastes, and isolation of thermostable enzymes.

Many factors affect the anaerobic metabolism of *Thermotogae* species, including operating conditions (shaking, inoculum, gas sparging, and culture/headspace volume ratio), temperature, pH, nitrogen, sulfur-containing compounds, sodium chloride, phosphate, and metal ions. Optimization of these fermentation parameters has been intensively pursued with *Thermotoga* and *Pseudothermotoga* species, which are the best hydrogen producers in the phylum. In contrast, little is known regarding other species of the phylum, especially their ability to synthesize desirable biological products.

In general, *Thermotogae* fermentation is affected by the accumulation of produced biogas and organic acids because they increase hydrogen partial pressure inside of the bioreactor and drastically reduce the pH of the cultivation medium. Consequently, the metabolic process stops before the substrate is completely consumed. Gas sparging, stirring, and adjusting culture/headspace volume ratio can help to overcome the inhibition on growth caused by hydrogen accumulation. Implementing these strategies and adjusting pH during the fermentation process can result in high hydrogen yields and efficient consumption of substrates. A reduction of fermentation time by starting with the right inoculum size could cast favorable great perspectives on the economics of the industrial processes.

This review highlights the importance of nitrogen-containing compounds that need to be supplied to the medium to stimulate bacterial growth. Overall, yeast extract and tryptone are the preferred forms of nitrogen. Sulfur-containing compounds not only play a critical role in bacterial growth but also divert reducing power to selectively produce certain end-products in *Thermotogae* metabolism.

Until now, the impact of metal ions and salts on the fermentation process has not been well investigated even though it has been demonstrated that they could stimulate many key enzymes involved in various metabolic pathways.

In summary, the extensive data collection of this review offers a great reference for the optimization and development of sustainable bioprocesses based on *Thermotogae* species and helps to generate insightful perspectives for the exploitation of these anaerobic bacteria in biotechnological processes.

## Figures and Tables

**Table 1:** Physiological and metabolic properties of *Thermotogae* species. **YE**: Yeast extract; **BHI**: Brain heart infusion; **CMC**: Carboxymethylcellulose; **S^0^** = Elemental sulfur; **Thio**: Thiosulfate; **Cys**: Cysteine; **AA**: Acetic acid; **LA**: Lactic acid; **ALA**: Alanine; **EPS**: Exopolysaccharide; **AABA**: α-aminobutyrate; **EtOH**: Ethanol; **AQDS**: Anthraquinone-2,6-disulfonate; **But**: Butyrate; **Val**: Valerate; **Glu**: Glutamate; **BuOH**: Butanol; **iBut:** isobutyrate; **iVal:** isovalerate; **PPA**: Propionic Acid; **Gly**: Glycine; **Pro**: Proline; **Fo**: Formate; **HPA**: Hydroxyphenilacetate; **PA**: Phenylacetate; **3-IAA**: Indole-3-acetate; **2-MeBu**: 2-Methylbutyrate.

Genus	Species	Isolation	Temp. Range/ Optimal (°C)	pH Range/ Optimal	Cell Dimension (Long by Wide) (µm)	Growth Substrates	NaCl Range/ Optimal (%)	Electron Acceptor	End Products	Ref.
***Thermotoga***	*Thermotoga petrophila*	Oil reservoir, Japan	47–88/ 80	5.2–9.0/ 7.0	2.0–7.0 by 0.7–1.0	YE, peptone, glucose, fructose, ribose, arabinose, sucrose, lactose, maltose, starch, cellulose	0.1–5.5/ 1.0	S^0^; Thio	AA, LA, CO_2_, H_2_	[72]
	*Thermotoga naphthophila*	Oil reservoir, Japan	48–86/ 80	5.4–9.0/ 7.0	2.0–7.0 by 0.8–1.2	YE, peptone, glucose, galactose, fructose, mannitol, ribose, arabinose, sucrose, lactose, maltose, starch	0.1–6.0/ 1.0	S^0^; Thio	AA, LA, CO_2_, H_2_	[72]
	*Thermotoga maritima*	Geotermal vent	55–90/ 80	5.5–9.0/ 6.5	1.5–11.0 by 0.6	ribose, xylose, glucose, sucrose, maltose, lactose, galactose, starch, glycogen	0.2–3.8/ 2.7	Fe (III) S^0^; Thio	AA, LA, CO_2_, H_2_, ALA, EPS, AABA	[3]
	*Thermotoga profunda*	Hot spring, Japan	50–72/ 60	6.0–8.6/ 7.4	0.8–2.1 by 0.4	glucose, trehalose, cellobiose, arabinose, xylose, ribose, pyruvate	n. d	S^0^; Thio	n. d	[73]
	*Thermotoga caldifontis*	Hot spring, Japan	55–85/ 70	6.0–8.6/ 7.4	1.2–3.5 by 0.5	glucose, maltose, trehalose, cellobiose, arabinose, xylose, ribose, pyruvate, starch	n. d	Thio	n. d	[73]
	*Thermotoga neapolitana*	Submarine thermal vent	55–95/ 77	6.0–9.0/ 7.5	1.5–11.0 by 0.6	fructose, fucose, galactose, mannose, rhamnose, pyruvate, glucosamine, lactulose, turanose, glycerol, dextrin, ribose, xylose, glucose, sucrose, maltose, lactose, starch, glycogen	0.2–6.0/ 2.0	S^0^	AA, ALA, CO_2_, H_2_	[74]
***Pseudothermotoga***	*Pseudothermotoga lettingae*	Thermophilic bioreactor	50–75/ 65	6.0–8.5/ 7.0	2.0–3.0 by 0.5–1.0	glucose, EtOH, acetate, formate	0.0–2.8/ 1.0	S^0^; Thio; AQDS; Fe(III)	AA, ALA, LA, EtOH, AA, BA, CO_2_, H_2_	[75]
	*Pseudothermotoga elfii*	Oil reservoir	50–72/ 66	5.5–7.5/ 7.5	2.0–3.0 by 0.5–1.0	glucose, arabinose, fructose, lactose, maltose, mannose, ribose, sucrose, xylose	0.0–2.8/ 1.0	Thio	AA, CO_2_, H_2_	[76]
	*Pseudothermotoga hypogea*	Oil reservoir, Africa	56–90/ 70	6.1–9.1/ 7.3–7.4	2.0–3.0 by 0.5–1.0	fructose, galactose, glucose, lactose, maltose, mannose, sucrose, xylose, xylan	0.0–1.5/ 0.2	Thio	AA, ALA, CO_2_, H_2_, EtOH	[77]
***Pseudothermotoga***	*Pseudothermotoga subterranea*	Oil reservoir, Paris	50–75/ 70	6.0–8.5/ 7.0	3.0–10.0 by 0.5	YE, peptone, tryptone, casein	0.0–2.4/ 1.2	Cys, Thio	n.d.	[78]
	*Pseudothermotoga thermarum*	Hot spring, Africa	55–84/ 70	6.0–9.0/ 7.0	1.5–11.0 by 0.6	starch, glucose, maltose	0.2–0.5/ 0.35	S^0^	n.d.	[6]
***Fervidobacterium***	*Fervidobacterium nodosum*	Hot spring, New Zealand	40–80/ 65–70	6.0–8.0/ 7.0	1.0–2.5 by 0.5–0.55	glucose, sucrose, starch and lactose	n.d./<1.0	S^0^	AA, LA, CO_2_, H_2_, EtOH, But, Val	[5]
	*Fervidobacterium pennavorans*	Hot spring, Portugal	50–80/ 70	5.5–8.0/ 6.5	2.0–20.0 by 0.5	cellobiose, starch, glycogen, pullulan, glucose, fructose, maltose, xylose, native feathers	0.0–4.0/ 0.4	S^0^; Thio	AA, CO_2_, ALA, Glu, EtOH, But, H_2_, BuOH	[79]
	*Fervidobacterium islandicum*	Icelandic Hot spring	50–80/ 65	6.0–8.0/ 7.2	1.0–4.0 by 0.6	pyruvate, ribose, glucose, maltose, raffinose, starch, cellulose	0.0–1.0/ 0.2	S^0^; Thio	LA, AA, H_2_, EtOH, CO_2_, iBut, iVal	[80]
	*Fervidobacterium riparium*	Hot spring, Russia	46–80/ 65	5.7–7.9/ 7.8	1.0–3.0 by 0.4–0.5	peptone, YE, pyruvate, glucose, xylose, fructose, maltose, sucrose, cellobiose, starch, xylan, CMC, cellulose, filter paper	0.0–1.0/ 0.0	S^0^	H_2_, AA, CO_2_, PPA, iBut, But	[81]
	*Fervidobacterium gondwanense*	Hot spring, Australia	45–80/ 65–68	5.5–8.5/ 7.0	4.0–40.0 by 0.5–0.6	cellobiose, amylopectin, maltose, starch, dextrin, xylose, glucose, pyruvate, lactose, fructose, mannose, CMC, galactose	0.0–0.6/ 0.1	S^0^	EtOH, AA, LA, CO_2_, H_2_	[82]
	*Fervidobacterium thailandese*	Hot spring, Thailand	60–88/ 78–80	6.5–8.5/ 7.5	1.1–2.5 by 0.5–0.6	glucose, maltose, sucrose, fructose, cellobiose, CMC, cellulose, starch	<0.5/0.5	S^0^	n.d.	[83]
	*Fervidobacterium changbaicum*	Hot spring, China	55–90/ 75–80	6.3–8.5/ 7.5	1.0–8.0 by 0.5–0.6	glucose, lactose, fructose, sucrose, maltose, starch, sorbitol, cellobiose, trehalose, galactose, melibiose, pyruvate, glycerin	0.0–1.0/ 0.0	S^0^	n.d.	[84]
***Thermosipho***	*Thermosipho* *africanus*	Hot spring, Africa	53–77/ 75	6.0–8.0/ 7.2	3.0–4.0 by 0.5	glucose, ribose, maltose, starch, galactose, fructose, sucrose	0.11–3.6	S^0^; Thio	AA, H_2_, CO_2_, EtOH, LA	[85]
	*Thermosipho* *japonicus*	Hydrothermal vent, Japan	45–80/ 72	5.3–9.3/ 7.2–7.6	3.0–4.0 by 0.5	YE, peptone, and tryptone, maltose, glucose, galactose, starch, sacharose, ribose, casein	0.7–7.9/ 4.0	S^0^; Thio	n.d.	[86]
	*Thermosipho* *geolei*	Oil reservoir, Russia	45–75/ 70	6.0–9.4/ 7.5	2.0–3.0 by 0.4–0.6	Glucose, peptone, beef extract, YE	0.5–7.0/ 2.0–3.0	S^0^	H_2_, AA, ALA, CO_2_, iVal	[87]
***Thermosipho***	*Thermosipho* *affectus*	Hydrothermal vent, Atlantic Ocean	37–75/ 70	5.6–8.2/ 6.6	1.2–6.0 by 0.4–0.9	YE, beef extract, glucose, maltose, sucrose, starch, dextrin, CMC, cellulose	1.0–5.5/ 2.0	S^0^	AA, H_2_, CO_2_, EtOH	[88]
	*Thermosipho* *globiformans*	Hydrothermal vent	40–75/ 68	5.0–8.2/ 6.8	2.0–4.0 by 0.5	YE, tryptone, starch	0.2–5.2/ 2.5	S^0^; Fe_2_O_3_	n.d.	[89]
	*Thermosipho* *melanesiensis*	Hydrothermal vent, Pacific Ocean	50–75/ 70	4.5–8.5/ 6.5–7.5	1.0–3.5 by 0.4–0.6	BHI, malt extract, tryptone, sucrose, starch, glucose, maltose, lactose, cellobiose, galactose	1.0–6.0/ 3.0	S^0^	H_2_, AA, ALA, CO_2_	[90]
	*Thermosipho* *activus*	Riftia sheath, Guaymas Basin	44–75/ 65	5.5–8.0/ 6.0	1.5–10.0 by 0.3–0.8	glucose, maltose, cellobiose, cellulose, filter paper, chitin, xylan, pectin, xanthan gum, YE, beef extract, tryptone, casein, keratin, arabinose, xylose, gelatin	0.3–6.0/ 2.5	S^0^, Fe (III)	AA, H_2_, CO_2_	[91]
	*Thermosipho* *atlanticus*	Hydrothermal vent, Atlantic Ocean	45–80/ 65	5.0–9.0/ 6.0	1.0–2.6 by 0.2–0.6	cellobiose, xylose, starch, LA, maltose, mannose, trehalose, lactose, arabinose, galactose, mannitol, peptone, casamino acids, gelatin, BHI, YE, glucose	1.5–4.6/ 2.3	S^0^, Thio, Cys	AA, iVal, H_2_, Gly, ALA, Pro	[92]
***Geotoga***	*Geotoga* *subterranea*	Oilfields, USA	30–60/ 45	5.5–9.0/ 6.5	4.0– 7.5 by 0.5	mannose, starch, maltodextrins, glucose, lactose, sucrose, galactose, maltose	0.5-10/ 4.0	S^0^	H_2_, CO_2_, AA, EtOH	[10]
	*Geotoga* *petraea*	Oilfields, USA	30–55/ 50	5.5–9.0/ 6.5	3.0– 20.0 by 0.6	mannose, starch, maltodextrins, glucose, lactose, sucrose, galactose, maltose	0.5–10/ 3.0	S^0^	H_2_, CO_2_, AA, EtOH	[10]
***Petrotoga***	*Petrotoga* *miotherma*	Oilfields, USA	35–65/ 55	5.5–9.0/ 6.5	2.0– 7.5 by 0.6	mannose, starch, maltodextrins, glucose, lactose, sucrose, galactose, maltose, maltodexstrins, xylose	0.5–10/ 2.0	S^0^	H_2_, CO_2_, AA, EtOH	[10]
	*Petrotoga* *olearia*	Oil reservoir, Russia	37–60/ 55	6.5–8.5/ 7.5	0.9–2.5 by 0.3–0.6	arabinose, xylose, cellobiose, dextrin, sucrose, glucose, fructose, maltose, ribose, trehalose, xylan, pyruvate, peptone, starch	0.5–8.0/ 2.0	S^0^	H_2_, AA, LA, ALA, EtOH	[93]
	*Petrotoga* *sibirica*	Oil reservoir, Russia	37–55/ 55	6.5–9.4/ 8.0	0.9–2.5 by 0.3–0.6	sucrose, glucose, fructose, maltose, ribose, trehalose, xylan, pyruvate, peptone, galactose	0.5–7.0/ 1.0	S^0^	H_2_, AA, LA, ALA, EtOH	[93]
***Petrotoga***	*Petrotoga* *mobilis*	Oilfield, North Sea	40–65/ 58–60	5.5–8.5/ 6.5–7.0	1.0–50.0 by 0.5–1.5	starch, xylan, maltodextrin, maltose, cellobiose, sucrose, lactose, glucose, galactose, fructose, arabinose, xylose, ribose, rhamnose	0.5–9.0/ 3.0–4.0	S^0^, Thio	H_2_, CO_2_, AA, EtOH	[94]
	*Petrotoga* *halophila*	Offshore oil, Africa	45–65/ 60	5.6–7.8/ 6.7–7.2	2.0–45.0 by 0.5–0.7	arabinose, cellobiose, fructose, galactose, glucose, lactose, maltose, rhamnose, ribose, starch, sucrose, xylose, xylan, pyruvate	0.5–9.0/ 4.0–6.0	S^0^	AA, LA, ALA, H_2_, CO_2_	[95]
	*Petrotoga* *mexicana*	Offshore oil, Africa	25–65/ 55	5.8–8.5/ 6.6	1.0–30.0 by 0.5–0.7	arabinose, cellobiose, fructose, galactose, glucose, lactose, maltose, mannose, raffinose, rhamnose, ribose, starch, sucrose, xylose, xylan, pyruvate.	1.0–20.0/ 3.0	S^0^, Thio, Sulfite	AA, LA, H_2_, CO_2_, ALA	[96]
	*Petrotoga* *japonica*	Oil reservoir, Japan	40–65/ 60	6.0–9.0/ 7.5	2.5–7.0 by 0.25–0.75	starch, xylan, maltose, cellobiose, sucrose, lactose, glucose, galactose, fructose, casamino acids, mannose, arabinose, xylose, ribose	0.5–9.0/ 0.5–1.0	S^0^, Thio	AA, H_2_, CO_2_, ALA	[97]
***Marinitoga***	*Marinitoga* *piezophila*	Hydrothermal chimney, Pacific Ocean	45–70/ 65	5.0–8.0/ 6.0	1.0–1.5 by 0.5	starch, fructose, glucose, galactose, maltose, cellobiose, ribose, acetate	1.0–5.0/ 3.0	S^0^, Thio, Cys	n.d.	[98]
	*Marinitoga* *litoralis*	Hot spring, Indian Ocean	45–70/ 65	5.5–7.5/ 6.0	1.0–7.0 by 0.8–1.0	cellobiose, galactose, glucose, glycogen, lactose, maltose, ribose, starch, BHI, casamino acids, casein, peptone, pyruvate, tryptone, YE	0.8–4.6/ 2.6	S^0^	n.d.	[99]
	*Marinitoga* *okinawensis*	Hydrothermal field, Okinawa	30–70/ 55–60	5.5–7.4/ 5.5–5.8	1.5–5.0 by 0.5–0.8	YE, tryptone, peptone, starch, glucose, glycerol	1.0–5.5/ 3.0–3.5	S^0^, Cys	n.d.	[100]
	*Marinitoga* *hydrogenitolerans*	Hydrothermal chimney, Atlantic Ocean	35–65/ 60	4.5–8.5/ 6.0	1.5–5.0 by 0.5–0.8	glucose, starch, glycogen, chitin, YE, BHI, peptone, casein, pyruvate, maltose	1.0–6.5/ 3.0–4.0	S^0^, Thio, Cys	AA, EtOH, Fo, H_2_, CO_2_	[101]
	*Marinitoga* *artica*	Hydrothermal chimney, Norwegian	45–70/ 65	5.0–7.5/ 5.5	1.0–5.0 by 0.5–0.8	glucose, trehalose, maltose, sucrose, maltodextrin, starch, pectin, meat extract, tryptone, YE, pyruvate, fructose, mannose, cellobiose, cellulose, peptone	1.5–5.5/ 2.5	S^0^, Cys	n.d.	[102]
	*Marinitoga* *camini*	Hydrothermal chimney, Atlantic Ridge	25–65/ 55	5.0–9.0/ 7.0	2.0–3.0 by 0.5–1.0	BHI, gluten, peptone, tryptone, pyruvate, glucose, fructose, maltose, cellobiose, sucrose, starch, cellulose, CMC, pectin, chitin	1.0–4.5/ 2.0	S^0^, Cys	AA, iBut, iVal, H_2_, 3-IAA, LA CO_2_, HPA, PA	[11]
***Oceanotoga***	*Oceanotoga* *teriensis*	Offshore oil, India	25–70/ 55– 58	5.5–9.0/ 7.5	1.5–1.7 by 0.5–0.7	glucose, fructose, cellobiose, arabinose, raffinose, rhamnose, sucrose, xylose, ribose, starch, EtOH, formate, acetate, BHI, YE, bio–trypticase	0.0–12/ 4.3	S^0^, Thio	AA, H_2_, CO_2_, EtOH	[12]
***Defluviitoga***	*Defluviitog* *tunisiensis*	Mesothermic digester	37–65/ 55	6.7–7.9/ 6.9	3.0–30.0 by 1.0	arabinose, cellobiose, fructose, galactose, glucose, lactose, maltose, mannose, raffinose, ribose, sucrose, xylose, cellulose, xylan	0.2–3.0/ 0.5	S^0^, Thio	AA, H_2_, CO_2_	[9]
***Mesotoga***	*Mesotoga* *infera*	Deep aquifer, France	30–50/ 45	6.2–7.9/ 7.4	2.0–4.0 by 1.0–2.0	arabinose, cellobiose, fructose, galactose, glucose, lactose, LA, mannose, maltose, raffinose, ribose, sucrose, xylose	0.0–1.5/ 0.2	S^0^	AA, CO_2_	[26]
	*Mesotoga* *prima*	Sediment, USA	20–50/ 37	6.5–8.0/ 7.5	1.0 by 0.2	xylose, fructose, ribose, sucrose, mannose, galactose, maltose, lactose, peptone, tryptone, casamino acids, glucose, arabinose, cellobiose, casein, pyruvate	2.0–6.0/ 4.0	S^0^, Thio, Sulfite	AA, But, iBut, iVal, 2–MeBu	[8]
***Kosmotoga***	*Kosmotoga* *arenicorallina*	Hot spring, Japan	50–65/ 60	6.2–8.0/ 7.1	1.1–2.7 by 1.1–1.9	xylose, maltose, glycerol	1.0–6.0/ 3.0	S^0^, Cys	n.d.	[103]
	*Kosmotoga* *pacifica*	Hydrothermal field, Pacific Ocean	33–78/ 70	6.2–8.0/ 7.1	1.0 by 0.6	maltose, YE, peptone, BHI, glycerol, tryptone, xylose, glucose, fructose, cellobiose, trehalose, LA, propionate, glutamate	0.5–6.0/ n.d.	S^0^, Cys	n.d.	[104]
	*Kosmotoga* *olearia*	Fluid, North Sea	20–80/ 65	5.5–8.0/ 6.8	0.8–1.2 by 0.4–0.7	maltose, ribose, sucrose, starch, casamino acids, tryptone, pyruvate	1.0–6.0/ 2.5–3.0	Thio	H_2_, CO_2_, AA, EtOH, PPA	[7]
	*Kosmotoga* *shengliensis*	Oilfield, China	45–75/ 65	6.0–8.0/ 7.0	0.7–0.9	glucose, acetate, mEtOH, galactose, fructose, xylose, sucrose, maltose, sorbitol, lactose, xylan, arabinose, formate, rhamnose, glycerol, pyruvate, starch, LA	0.0–4.0/ 1.5	S^0^, Thio, Sulfate	AA, LA, ALA, CO_2_, H_2_	[15]
***Athalassatoga***	*Athalassatoga* *saccharophila*	Hot spring, Japan	30–60/ 55	4.5–7.5/ 5.5–6.0	0.8–2.0 by 0.7–0.8	arabinose, fructose, glucose, lactose, maltose, mannose, ribose, sucrose, xylose, starch, glycogen, peptone, YE	<1/0.0	Fe (III), Thio, Cys	AA, iBut, iVal	[14]
***Mesoaciditoga***	*Mesoaciditoga* *lauensis*	Hydrothermal vent, Pacific Ocean	45–65/ 57–60	4.1–6.0/ 5.5–5.7	0.8–1.0 by 0.4	YE, peptone, maltose, sucrose, glucose, xylose, ribose, starch, tryptone	0.5–6.0/ 3.0	S^0^; Thio, Cys	n.d.	[13]

**Table 2:** Effects of operating conditions on *Thermotogae* fermentation. MOPS: Morpholinopropane-1-sulfonic acid; **HEPES**: 2-[4-(2-hydroxyethyl) piperazin-1-yl] ethanesulfonic acid; **TRIS**: tris(idrossimetil)amminometano cloridrato; **CDW**: Cellular dry weight; **AA**: Acetic acid; **LA**: Lactic acid; **ALA**: Alanine; **But**: Butyrate; **IA**: Itaconic acid; GaR: recirculation of H_2_-rich biogas. Experiments were performed in different bioreactor configurations: **B** = Batch; **CSTR** = Continuous-flow Stirred-Tank Reactor; **CSABR**: Continuously Stirred Anaerobic Bioreactor; **SB** = Serum bottles. **H_2_ column**: ^a^ H_2_ yield = mol H_2_/mol consumed substrate; ^b^ mL/L culture. * Values extrapolated from the graphical representation of data.

Parameter	Organism	T (°C)	Culture Type	Mixing Speed (rpm)	Reactor/Working Volume (L)	Substrate Loaded (mmol/L)	Operational Parameter	Substrate Consumed (mmol/L)	Products					Ref.
									H_2_ yield^a^	AA (mmol/L)	LA (mmol/L)	ALA (mmol/L)	But (mmol/L)	
***P*_H2_ (mbar)**	*T.* *maritima*	80	B	350	1.4/0.1	Glucose (28)	*P*_H2_ = 7.1 ± 0.4	19.8 ± 1.1	2.34	25.0 ± 1.4	10.5 ± 0.5			[107]
							*P*_H2_ = 71.4 ± 2.1	19.7 ± 1.4	2.44	24.6 ± 2.4	11.0 ± 0.6			
							*P*_H2_ = 178.5 ± 3.5	17.2 ± 0.9	2.32	20.1 ± 1.0	9.4 ± 0.5			
							*P*_H2_ = 606.9 ± 18.7	13.4 ± 0.7	n. d.	13.0 ± 0.7	11.0 ± 0.6			
**Stirring Speed (rpm)**	*T.* *neapolitana*	75	CSABR	300	3.0/1.0	Xylose (33.3)	300	31.43	2.13 ± 0.11	41.8 ± 2.16	1.78 ± 0.11			[113]
				400			400	32.56	2.94 ± 0.15	50.12 ± 2.5	4.0 ± 0.22			
				500			500	32.03	2.31 ± 0.12	44.62 ± 2.16	4.84 ± 0.22			
				600			600	31.87	2.24 ± 0.11	41.12 ± 2.0	1.89 ± 0.11			
	*T.* *neapolitana subsp.* *capnolactica*	80	CSTR	300	3.0/2.0	Glucose (28)	300	22.9 ± 2.7	3.0 ± 0.0	32.3 ± 4.3	10.0 ± 1.0	1.1 ± 0.1		[69]
				500			500	24.8 ± 0.4	3.2 ± 0.1	37.7 ± 2.7	8.1 ± 0.2	1.0 ± 0.1		
				300			300 + GaR	24.7 ± 0.2	3.5 ± 0.2	39.2 ± 1.2	4.4 ± 0.1	0.9 ± 0.0		
				500			500 + GaR	24.9 ± 0.2	3.3 ± 0.1	38.7 ± 2.2	5.1 ± 0.5	0.8 ± 0.0		
**Gas sparging**	*T.* *neapolitana*	80	B	250	3.8/1.0	Glucose (28)	N_2_	25.9 ± 1.3	2.8	44.8 ± 5.4	12.5 ± 2.9	1.3 ± 0.4		[31]
							CO_2_	26.1 ± 1.2	2.8	35.6 ± 5.8	20.0 ± 6.1	2.7 ± 0.5		
		75	SB	no	0.12/0.04	Glycerol (108.6)	w/o	13 ±0.6	1.24 ± 0.06	8.71 ± 0.35	0.36 ± 0.02			[115]
							N_2_	14 ± 0.7	2.06 ± 0.09	10.04 ± 0.5	0.34 ± 0.02			
							N_2_ plus pH control	18 ± 0.9	1.98 ± 0.1	12.62 ± 0.53	0.25 ± 0.01			
**Gas** **sparging**	*T.* *neapolitana*	77	SB	150	0.12/0.04	Glucose (39)	w/o	-	1.82 ± 0.09	64.28 ± 2.83			33.48 ± 1.47	[110]
							N_2_	-	3.24 ± 0.14	81.42 ± 3.49			36.77 ± 2.04	
						Xylose (27)	w/o	-	1.14 ± 0.07	40.30 ± 3.5			37.68 ± 1.7	
							N_2_	-	2.20 ± 0.13	71.94 ± 3.66			50.62 ± 2.38	
	*T.* *neapolitana subsp.* *capnolactica*	80	SB	no	0.12/0.03	Glucose (28)	N_2_	25.7 ± 0.1	2.5 ± 0.06	27.3 ± 0.8	8.6 ± 0.2	2.5 ± 0.2		[70]
							CO_2_	28.3 ± 1.0	2.9 ± 0.1	22.1 ± 0.9	11.3 ± 0.1	3.0 ± 0.3		
	*T.* *neapolitana*	80	SB	no	0.12/0.03	Glucose (28)	N_2_	21.7 ± 0.6	2.5 ± 0.03	30.2 ± 0.4	2.2 ± 0.02	1.9 ± 0.3		
							CO_2_	20.8 ± 2.3	1.9 ± 0.1	20.8 ± 0.1	1.2 ± 0.06	2.4 ± 0.3		
	*T.* *maritima*	80	SB	no	0.12/0.03	Glucose (28)	N_2_	23.2 ± 1.0	1.9± 0.06	25.5 ± 0.5	5.3 ± 0.8	2.4 ± 0.06		
							CO_2_	19.9 ± 0.6	2.0 ± 0.1	18.3 ± 0.3	1.6 ± 0.2	2.3 ± 0.3		
	*T.* *naphtophila*	80	SB	no	0.12/0.04	Glucose (28)	N_2_	13.30 ± 1.10	2.20 ± 0.20	15.70 ± 0.10	1.40 ± 0.06	0.80 ±0.10		
							CO_2_	20.80 ± 1.70	1.60 ± 0.20	19.20 ± 0.10	5.00 ± 0.02	1.80 ±0.05		
	*T.* *petrophila*	80	SB	no	0.12/0.05	Glucose (28)	N_2_	9.20 ± 1.30	3.00 ± 0.40	13.10 ± 0.05	2.00 ± 0.01	0.00		
							CO_2_	14.20 ± 0.60	1.90 ± 0.10	12.60 ± 0.10	3.80 ± 0.02	0.30 ±0.10		
	*T.* *caldifontis*	70	SB	no	0.12/0.05	Glucose (28)	N_2_	10.90 ± 1.10	2.60 ± 0.10	16.70 ± 3.60	2.20 ± 0.50	3.20 ±0.90		
							CO_2_	15.20 ± 0.90	1.80 ± 0.03	15.60 ± 1.50	2.30 ± 0.40	6.60 ±0.70		
	*T.* *profunda*	60	SB	no	0.12/0.05	Glucose (28)	N_2_	18.1 0 ±0.40	1.50 ± 0.20	15.90 ± 0.40	5.70 ± 0.10	1.40 ±0.06		
							CO_2_	22.60 ± 1.70	0.70 ± 0.04	5.60 ± 0.20	2.3 ± 0.04	2.60 ±0.30		
	*Pseudot. hypogea*	70	SB	no	0.12/0.05	Glucose (28)	N_2_	8.80 ± 1.10	1.10 ± 0.30	6.40 ± 0.10	0.10 ± 0.00	2.90 ±0.10		
							CO_2_	4.30 ± 0.10	0.50 ± 0.10	3.10 ± 0.20	0.10 ± 0.00	3.40 ±0.30		
	*Pseudot. elfii*	70	SB	no	0.12/0.05	Glucose (28)	N_2_	7.00 ± 0.90	2.00 ± 0.20	8.30 ± 0.06	0.20 ± 0.03	4.20 ±0.30		[70]
							CO_2_	6.70 ± 0.20	2.10 ± 0.10	7.80 ± 0.30	0.10 ± 0.01	10.0 ±0.30		
	*Pseudot. lettingae*	70	SB	no	0.12/0.05	Glucose (28)	N_2_	9.30 ± 0.50	1.20 ± 0.10	5.10 ± 0.05	0.20 ± 0.00	2.70 ±0.05		
							CO_2_	8.10 ± 0.70	1.30 ± 0.30	4.40 ± 0.10	0.05 ± 0.01	3.70 ±0.20		
**Gas** **sparging**	*Pseudot. subterranea*	70	SB	no	0.12/0.05	Glucose (28)	N_2_	23.10 ± 2.10	1.80 ± 0.20	30.60 ± 6.90	16.20 ± 4.60	9.50 ±0.40		
							CO_2_	27.00 ± 1.40	1.40 ± 0.10	31.90 ± 7.90	10.70 ± 4.0	20.0 ± 8.0		
	*Pseudot. thermarum*	80	SB	no	0.12/0.05	Glucose (28)	N_2_	Complete	1.8 ± 0.02	30.00 ± 2.20	6.50 ± 0.20	1.10 ±0.07		
							CO_2_	Complete	1.50 ± 0.10	24.80 ± 0.70	5.60 ± 0.60	2.20 ±0.20		
**Biomass** **(g CDW/L)**	*T.* *neapolitana*	80	Flask	300	0.25/0.2	Glucose (28)	0.46	3.2 ± 0.04	2.39	34.3 ± 0.6	10.9 ± 0.4			[68]
							0.91	2.9 ± 0.06	2.44	32.9 ± 0.8	12.2 ± 0.8			
							1.33	3.4 ± 0.01	2.58	32.3 ± 0.2	11.5 ± 0.5			
							1.74	3.0 ± 0.04	2.37	31.4 ± 1.1	14.7 ± 0.7			
**pH**	*T.* *neapolitana subsp.* *capnolactica*	80	SB	no	0.12/0.03	Glucose (28)	w/o	18.54 ± 0.15	1.78 ± 0.29	22.76 ± 0.40	11.35 ± 0.62			[67]
							0.01M MOPS	26.42 ± 0.05	3.27 ± 0.18	26.65 ± 0.87	14.23 ± 0.22			
							0.01M TRIS	25.55 ± 0.06	3.10 ± 0.10	26.77 ± 0.29	12.08 ± 0.89			
							0.01M HEPES	25.99 ± 0.03	2.85 ± 0.40	25.56 ± 0.49	13.58 ± 0.88			
							0.01M HCO_3_^−^	25.62 ± 0.10	2.20 ± 0.30	22.82 ± 0.84	14.63 ± 3.23			
							0.01M phosphate	26.17 ± 0.26	2.78 ± 0.40	24.70 ± 0.59	14.92 ± 0.25			
	*T.* *neapolitana*	75	CSABR	300	3.0/1.0	Glucose (28)	w/o pH control	21.98 ± 1.11	2.05 ± 0.1	30.81 ± 1.5	3.33 ± 0.22			[113]
							plus pH control	27.47 ± 1.39	3.2 ± 0.16	38.3 ± 2.0	1.77 ± 0.11			
						Xylose (33.3)	w/o pH control	29.77 ± 1.46	1.84 ± 0.09	34.47 ± 1.66	3.77 ± 0.22			
							plus pH control	31.83 ± 1.6	2.22 ± 0.11	41.8 ± 2.0	1.66 ± 0.11			
**pH**	*T.* *neapolitana*	75	CSABR	300	3.0/1.0	Sucrose (14.6)	w/o pH control	13.78 ± 0.7	3.52 ± 0.18	33.13 ± 1.65	3.11 ± 0.11			[113]
							plus pH control	14.69 ± 0.06	4.95 ± 0.25	35.47 ± 1.83	2.11 ± 0.11			
						Xylose (33.3)	w/o pH control	29.44	1.85 ± 0.09	34.97 ± 1.66	3.88 ±0.22			
							pH = 6.5	32.57	2.71 ± 0.14	49.62 ± 2.50	3.44 ± 0.11			
							pH = 7.0	32.9	2.84 ±0.14	50.29 ± 2.50	4.00 ± 0.22			
							pH = 7.5	31.77	2.23 ± 0.11	41.96 ± 2.16	1.89 ± 0.11			
		75	SB	no	0.04/ 0.12	Glycerol (108.6)	w/o HEPES	16.96 ± 0.8	1.23 ± 0.06	9.14 ± 0.45				[116]
							0.05 M HEPES	28.26 ± 1.4	2.73 ± 0.14	22.35 ± 1.05				
	*T.* *neapolitana*	80	B	250	3.8/1.0	Glucose (28)	w/o NaHCO_3_	25.9 ± 1.3	2.8	44.5 ± 5.4	12.5 ± 2.69			[31]
							NaHCO_3_ 14 mM	25.4 ± 2.1	1.7	30.5 ± 4.9	18.0 ± 0.6			
							NaHCO_3_ 20 mM	23.2 ± 1.9	1.0	44.4 ± 8.2	9.2 ± 2.7			
**pH**							NaHCO_3_ 40 mM	6.2 ± 0.8	2.7	18.0 ± 4.3	0.7 ± 1.5			
		75	B	no	0.12/0.04	Glycerol (108.6)	w/o IA	-	438 ± 22 ^b^	7.49 ± 0.33	3.55 ± 0.22 *			[122]
							1.5 g/L IA	-	619 ± 30 ^b^	11.49 ± 0.5	1.66 ± 0.0 *			
**Temp.** **(°C)**	*T.* *neapolitana*	60	SB	75	0.26/0.05	Glucose (14)	60	2.2 *	2.04 ± 0.05	2.0	n. d			[65]
		65					65	5.0 *	3.09 ± 0.3	7.0	0.05			
		70					70	8.5 *	3.18 ± 0.02	11.5	0.45			
		77					77	11.0 ± 0.5 *	3.85 ± 0.28	16.5	0.85 ± 0.1			
		85					85	11.0 ± 0.5 *	3.75 ± 0.49	18.0 ± 1.0	1.25 ± 0.05			
**Oxygen**	*T.* *maritima*	80	B	150	2.30/1.53	Glucose (20)	w/o O_2_	17.41	38.09 ^b^	18.05	4.36	1.60 ± 0.2		[129]
							with O_2_	19.30	31.75 ^b^	18.27	5.45	1.30 ± 0.2		

**Table 3:** Effect of organic nitrogen source and NaCl on *Thermotogae* fermentation. **AA**: Acetic acid; **LA**: Lactic acid; **ALA**: Alanine; **YE**: Yeast extract; **Tryp**: Tryptone; **CA**: Casamino acids; **V**: Vitamins solution [108]; **aa**: Amino acids (cysteine, alanine, asparagine, proline, glutamine, serine, and tryptophan, added at 0.2 g/L each). Experiments were performed in different bioreactor configurations: **B** = Batch; **SB** = Serum bottles. **H_2_ column**: ^a^ % H_2_ = calculated setting hydrogen production yield on medium with yeast extract to 100%; ^b^ mmol H_2_/L medium; ^c^ mL H_2_/L culture; ^d^ mol H_2_/mol glucose. * Values extrapolated from the graphical representation of data.

Parameter	Organism	T (°C)	Culture Type	Mixing Speed (rpm)	Reactor/Working Volume (L)	Substrate Loaded (mmol/L)	Operational Parameter	Substrate Consumed (mmol/L)	Products				Ref.
									H_2_	AA (mmol/L)	LA (mmol/L)	ALA (mmol/L)	
**Nitrogen sources (g/L)**	*Pseudot.* *elfii*	65	B	100	3.0/1.0	no	w/o YE	-	40 ^a^				[108]
							CA + V	-	4 ^a^				
							CA + V + aa	-	6 ^a^				
		65	B	100	3.0/1.0	Glucose (22.4)	YE (5)	n.d.	100 ^a^				
							CA + V	n.d.	14 ^a^				
							CA +V + aa	n.d.	14 ^a^				
		65	B	100	3.0/1.0	no	YE (2) -Tryp (0)	-	13.9 ^b^	3.5			
							YE (2) -Tryp (2)	-	14.8 ^b^	3.4			
							YE (5) -Tryp (0)	-	14.0 ^b^	0.0			
							YE (5) -Tryp (5)	-	28.8 ^b^	4.9			
		65	B	100	3.0/1.0	Glucose (56)	YE (2) -Tryp (0)	10.3	25.8 ^b^	10.7			
							YE (2) -Tryp (2)	18.3	78.5 ^b^	19.7			
							YE (5) -Tryp (0)	13.1	84.9 ^b^	26.3			
							YE (5) -Tryp (5)	17.9	82.5 ^b^	21.2			
	*T.* *neapolitana*	80	SB	no	0.12/0.05	Glucose (28)	YE (0.5)	26.6 *	260 *^c^	15 *			[64]
							YE (1.0)	26 *	320 *^c^	22.5 *			
							YE (2.0)	25.5 *	360 *^c^	26.6 *			
							YE (4.0)	25 *	430 *^c^	30 *			
							YE (6.0)	25 *	430 *^c^	33.3 *			
	*T.* *maritima*	80	SB	no	0.12/0.05	Glucose (28.00)	YE (0.5)	25.5 *	190 *^c^	0.0 *			
							YE (1.0)	25 *	260 *^c^	20.8 *			
							YE (2.0)	25 *	270 *^c^	23 *			
**Nitrogen sources (g/L)**	*T.* *maritima*	80	SB	no	0.12/0.05	Glucose (28.00)	YE (4.0)	25 *	335 *^c^	27.5 *			[64]
							YE (6.0)	24 *	390 *^c^	28 *			
	*T.* *neapolitana*	77	B	75	0.12/0.05	Glucose (28)	no YE	23 *	9 *^b^	4.2 *			[136]
							YE (0.5)	Completed *	16 *^b^	7.2 *			
**NaCl (g/L)**	*T.* *neapolitana subsp.* *capnolactica*	80	SB	no	0.12/0.03	Glucose (28)	w/o	25.62 ± 0.07	2.30 ± 0.50 ^d^	20.66 ± 0.27	2.80 ± 0.26	1.28 ± 0.9	[67]
							NaCl (5)	26.00 ± 0.14	2.50 ± 1.20 ^d^	24.59 ± 0.95	6.23 ± 3.26	1.61 ± 0.58	
							NaCl (10)	26.12 ± 0.16	3.10 ± 0.80 ^d^	26.05 ± 4.69	11.61 ± 2.42	2.46 ± 0.24	
							NaCl (20)	25.96 ± 0.11	3.30 ± 0.20 ^d^	25.58 ± 1.03	13.44 ± 0.94	2.41 ± 0.09	
							NaCl (30)	25.68 ± 0.25	2.91 ± 0.37 ^d^	23.22 ± 0.81	21.63 ± 6.15	2.38 ± 0.10	

**Table 4:** : Effect of sulfur compounds on *Thermotogae* fermentation. **AA**: Acetic acid; **LA**: Lactic acid; **ALA**: Alanine; **EtOH**: Ethanol; **iVal**: isovalerate; **H_2_S**: Hydrogen sulfide; **Glu**: Glutamate; **DMSO**: Dimethyl Sulfoxide; **S^0^**: Elemental sulfur; **Met**: Methionine; **Thio**: Thiosulfate; **Cys**: Cysteine; **Na_2_S**: Sodium sulfide. * Values extrapolated from the graphical representation of data. ** Concentrations of Sulfur compounds are 0.03 mol equivalent of sulfur. ^a^ H_2_ produced millimolar equivalent; ^b^ mmol; ^c^ µM.

Organism	Carbon Source (mM)	Sulfur Source (mM)	Substrate Consumed (mmol/L)	Products mmol/L Culture								Ref.
				H_2_	AA	LA	ALA	EtOH	iVal	H_2_S	Glu	
*T.* *maritima*	Glucose (25)	w/o	7.1 ± 0.4	21.3 ± 2.1	10.1 ± 0.8	0.8 ± 0.1	-					[107]
		DMSO **	9.2 ± 0.5	28.7 ± 2.9	13.3 ± 1.1	0.8 ± 0.1	-					
		S^0^ **	16.6 ± 0.8	46.1 ± 4.6	23.8 ± 1.9	3.4 ± 0.3	-					
		Met **	18.3 ± 0.9	53.3 ± 5.3	26.5 ± 2.1	3.1 ± 0.3	-					
		Thio **	17.5 ± 0.9	47.3 ± 4.7	24.1 ± 1.9	6.3 ± 0.6	-					
		Cys **	20.4 ± 1.0	58.5 ± 5.8	30.5 ± 2.4	4.1 ± 0.4	-					
		Na_2_S **	20.4 ± 1.0	54.9 ± 5.5	30.7 ± 2.5	4.7 ± 0.5	-					
	Glucose (60)	w/o Thio	17.7 ± 1.9	25.0 ± 2.2	12.8 ± 1.0	5.4 ± 0.6	1.39 ± 0.2					
		Thio (0.01)	20.0 ± 1.1	31.0 ± 2.3	16.0 ± 0.8	10.2 ± 1.1	-					
		Thio (0.03)	28.0 ± 1.5	57.9 ± 4.8	30.6 ± 1.9	8.2 ± 0.7	-					
		Thio (0.06)	38.5 ± 2.0	73.3 ± 5.9	38.2 ± 2.4	18.1 ± 1.8	-					
		Thio (0.12)	45.7 ± 2.5	99.7 ± 8.3	52.4 ± 3.3	15.4 ± 1.6	3.8 ± 0.3					
		Thio (0.18)	45.4 ± 2.2	86.9 ± 8.2	45.0 ± 2.2	23.4 ± 2.3	-					
		Thio (0.24)	43.8 ± 2.2	88.6 ± 8.9	46.1 ± 3.3	26.4 ± 1.4	3.8 ± 0.2					
	Glucose (20)	w/o	13.70	36.09	15.62		0.70			n.d.		[145]
		Thio (20)	13.55	4.02	15.99		0.80			14.45		
*T.* *neapolitana*	Glucose (20)	w/o	14.00	31.67	18.27		0.87			n.d.		[145]
		Thio (20)	13.90	16.07	16.12		0.60			7.39		
*Pseudot.* *lettingae*	Methanol (20)	w/o	19.70	n. d.	13.70		-			-		[75]
		Thio (20)	18.7	n. d.	-		5.8			11.2		
		S^0^ (2%)	10.6	n. d.	-		3.1			7.3		
*Pseudot.* *hypogea*	Glucose (20)	w/o	8.60	29.03	4.49		1.71			n. d.		[145]
		Thio (20)	14.39	2.29	19.7		1.06			15.08		
*Pseudot.* *hypogea*	Glucose (20)	w/o	7.0	9.4 ^a^	5.0		1.7	1.0		0.2		[77]
		Thio (20)	13.0	0.9 ^a^	19.8		1.0	1.6		15.1		
*Pseudot.* *hypogea*	Xylose (20)	w/o	12.9	19.0 ^a^	8.9		2.4	1.0		0.2		[77]
		Thio (20)	12.0	1.8 ^a^	13.7		1.3	1.0		7.5		
*Pseudot.* *elfii*	Glucose (20)	w/o	3.1	8.8	4.0					0.0		[77]
		Thio (20)	10.4	2.0	17.9					23.00		
	Glucose (20)	w/o	2.75	7.70	3.49		1.05			n. d.		[145]
		Thio (20)	8.15	n. d.	12.63		0.41			14.55		
*Ts.* *geolei*	Glucose (0.28)	w/o	7.0 ^b^	9.3 ^a^	8.5 ^b^		1.2 ^b^			0.5 ^b^		[87]
		S^0^ (2%)	6.0 ^b^	0.0 ^a^	7.5 ^b^		0.5 ^b^			12.5 ^b^		
*Ms.* *Prima Phos Ac3*	Glucose (20)	w/o	1.50 ± 0.20	<1 ^c^	1.67 ± 0.21					1.05 ± 0.25		[27]
		S^0^	6.57 ± 0.19	<1 ^c^	9.21 ± 0.13					24.40 ± 0.30		
*Ms.* *Prima MesG1Ag4.2T*	Fructose (20)	w/o	1.00 ± 0.23	<1 ^c^	0.70 ± 0.41					1.18 ± 0.41		
		S^0^	3.27 ± 0.85	<1 ^c^	8.48 ± 1.96					18.03 ± 5.16		
*Ts.* *africanus*	Glucose (28)	w/o	7.20	16.80	7.90	<0.2		0.79		n.d.		[145]
		Thio (20)	7.70	1.00	12.40	-		-		14.60		
*Ts.* *atlanticus*	Glucose (28)	w/o	5.6	12.5	1.7				0.14	-		[92]
		S^0^ (1%)	6.0	7.5	1.9				0.15	1.3		
*F.* *islandicum*	Glucose (20)	w/o	14.20	21.58	6.25		3.98			n.d.		[145]
		Thio (20)	16.20	n. d.	20.25		1.22			34.02		
*F.* *pennavorans*	Glucose (11)	w/o	-	0.25 *	6.7 *		4.0 ± 0.5 *				1.3 *	[32]
		Thio (20)	-	0.2 *	6.7 *		4.50 *				No *	
